# Cardioprotective Effect of *Centaurea castriferrei* Borbás & Waisb Extract against Doxorubicin-Induced Cardiotoxicity in H9c2 Cells

**DOI:** 10.3390/molecules28010420

**Published:** 2023-01-03

**Authors:** Ewelina Humeniuk, Grzegorz Adamczuk, Joanna Kubik, Kamila Adamczuk, Aleksandra Józefczyk, Agnieszka Korga-Plewko

**Affiliations:** 1 Independent Medical Biology Unit, Faculty of Pharmacy, Medical University of Lublin, 8b Jaczewski Street, 20-093 Lublin, Poland; 2 Department of Biochemistry and Molecular Biology, Faculty of Medical Sciences, Medical University of Lublin, 20-090 Lublin, Poland; 3 Department of Pharmacognosy with Medicinal Plant Laboratory, Faculty of Pharmacy, Medical University of Lublin, 1 Chodzki Street, 20-090 Lublin, Poland

**Keywords:** *Centaurea castriferrei* Borbás & Waisb, Asteraceae, doxorubicin, cardiomyocytes, cardiotoxicity

## Abstract

Doxorubicin (DOX) is one of the most used chemotherapeutic agents in the treatment of various types of cancer. However, a continual problem that is associated with its application in therapeutic regimens is the development of dose-dependent cardiotoxicity. The progression of this process is associated with a range of different mechanisms, but especially with the high level of oxidative stress. The aim of the study was to evaluate the effects of the water and methanol–water extracts from the plant *Centaurea castriferrei* (CAS) obtained by the ultrasound-assisted extraction method on the DOX-induced cardiotoxicity in the rat embryonic cardiomyocyte cell line H9c2. The H9c2 cells were treated for 48 h with the DOX and water or methanol–water extracts, or a combination (DOX + CAS H_2_O/CAS MeOH). The MTT assay, cell cycle analysis, and apoptosis detection revealed that both the tested extracts significantly abolished the cytotoxic effect caused by DOX. Moreover, the detection of oxidative stress by the CellROX reagent, the evaluation of the number of AP sites, and the expressions of the genes related to the oxidative stress defense showed substantial reductions in the oxidative stress levels in the H9c2 cells treated with the combination of DOX and CAS H_2_O/CAS MeOH compared with the DOX administered alone. The tested extracts did not affect the cytotoxic effect of DOX on the MCF-7 breast cancer cell line. The obtained results constitute the basis for further research in the context of the application of *C. castriferrei* extracts as adjuvants in the therapy regiments of cancer patients treated with DOX.

## 1. Introduction

A common side effect that is associated with the use of one of the most well-known chemotherapeutic agents, doxorubicin (DOX), is the development of cardiotoxicity. Many cancer patients develop cardiac complications that can lead to lifestyle disabilities or death [1,2,3]. Treatment schemes based on anthracyclines, including DOX, are still the mainstay of several anticancer treatment regimens, despite the significant advances in cancer therapy [4]. The anticancer activity of DOX is mainly based on the intercalation into the DNA structure and the inhibition of the topoisomerase II enzyme in rapidly proliferating cancer cells [5]. The drug’s mechanism of action generates excessive ROS and oxidative stress, acting strongly against the mitochondria and cellular DNA, and mainly in the tissues that are rich in these organelles: the heart, kidney, and liver [6,7]. DOX continues to play a significant role in the treatment of cancer; however, it causes cumulative and dose-dependent cardiotoxicity, which results in an increased risk of mortality among oncology patients, which thus limits its widespread clinical use [5,8,9,10]. In addition to oxidative stress, the main mechanisms that lead to cardiomyopathy are an impaired mitochondrial function, the disruption of Ca^2+^ homeostasis, inflammation, a perturbation in the iron regulatory protein, and apoptosis [5].

It is known that DOX-induced cardiomyopathy has a poor prognosis and can often lead to death [11]. Therefore, the search for cardioprotective therapies to minimize the effects of DOX treatment is reasonable. By using combination chemotherapy (the administration of DOX with other chemoprotective agents), the side effects caused by DOX can be overcome. This contributes to a reduction in the toxicity to healthy tissues and increases the effectiveness of doxorubicin [3,12,13].

Currently, there is a worldwide search for new plant species with anticancer activities that can provide an alternative to the classical chemotherapeutics due to the progressive resistance of cancer cells to the applied treatment [14,15,16]. In addition, much research is being conducted in the search for plant substances that have promising protective effects against the organs that are toxic to applied chemotherapy [3].

Over the past several years, there has been growing interest in the plants of the genus Centaurea L. (Asteraceae), which is a genus with several hundred representatives and that is native to the Mediterranean basin but is practically widespread on most continents [17,18]. The plants are quite expansive and do not require special soil conditions [19,20,21]. The genus Centaurea L. is currently being studied for its medicinal properties and potential use. These plants have valuable substances with strong antioxidant potential [17,22,23].

One of the least known and described plants of the genus Centaurea L. is *Centaurea castriferrei* Borbás & Waisb (CAS). This is a strongly endemic species that is native to the Vas region of Hungary [24]. In a recent study carried out by our group, we analyzed the phytochemical, antioxidant, and anticancer properties of the methanol–water (7:3 *v*/*v*) and water extracts of *C. castriferrei.* All the tested extracts showed antioxidant properties, but higher levels were noted in the case of the methanol–water extracts [16]. The obtained results indicate the high potential of *C. castriferrei* extracts to protect cardiomyocytes against the toxic effects of DOX-related oxidative stress. Therefore, the aim of the study was to evaluate the effect of water (CAS H_2_O) and methanol–water (CAS MeOH) extracts from the plant *C. castriferrei* obtained by the ultrasound-assisted extraction method on the DOX-induced cardiotoxicity in the rat embryonic cardiomyocyte cell line H9c2.

## 2. Results

### 2.1. Phytochemical Contents of CAS Extracts

The qualitative and quantitative analyses of the CAS MeOH and CAS H_2_O extract compositions were conducted using LC/(-)ESI–QTOK–MS and RP-HPLC/DAD, respectively. The results of the analyses are shown in Table 1, Table 2 and Table 3. The analyses revealed that both CAS MeOH and CAS H_2_O contain many active compounds, such as apigenin and its derivatives, chlorogenic acid, jacein, luteolin, centaurein, and others. Larger amounts and contents of active ingredients were noted in the case of the CAS MeOH extract.

### 2.2. Effects of CAS Extracts on DOX-Induced Toxicity in H9c2 Cells

**Figure 1:**
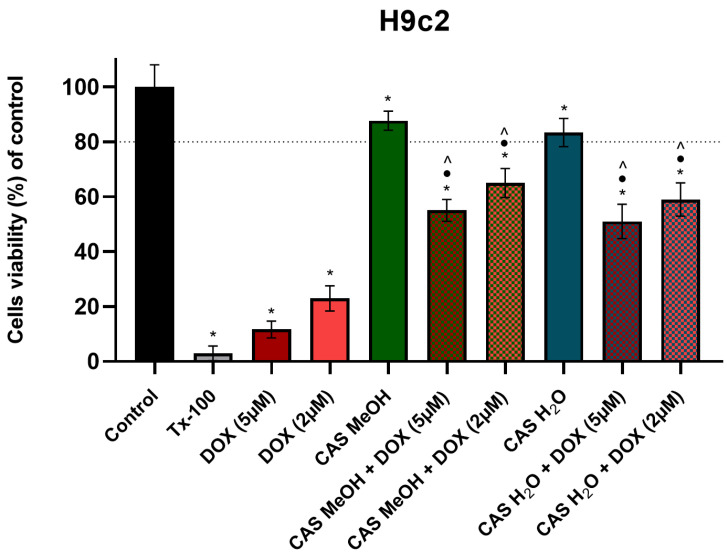
The H9c2 cell viability (% of control) based on MTT assay. Cells were treated for 48 h with 5 μM and 2 μM of DOX and 125 μg/mL of CAS H_2_O or 125 μg/mL of CAS MeOH, or a combination (DOX + CAS MeOH/DOX + CAS H_2_O). In addition, cells were treated with DMSO as a vehicle in control cultures and 1% Tx-100 in media as a positive control for 48 h. The values obtained from three independent experiments are presented as means ± SDs. * *p* < 0.05 vs. control; • *p* < 0.05 vs. DOX 5 µM/DOX 2 µM; ^ *p* < 0.05 vs. CAS MeOH/CAS H_2_O. Tx-100: Triton-x100; DOX: doxorubicin; CAS MeOH: *Centaurea castriferrei* Borbás & Waisb methanol–water (7:3 *v*/*v*) extract; CAS H_2_O: *Centaurea castriferrei* Borbás & Waisb water extract.

The cytotoxicity analysis performed with the MTT test indicated that 48 h of incubation at DOX concentrations of 5 µM and 2 µM caused statistically significant reductions in the viability of the H9c2 cells (to 10 ± 3.05% and 23 ± 4.58%, respectively) compared with the control cultures (Figure 1). The treatments with CAS H_2_O and CAS MeOH alone only marginally decreased the viability of the studied cells. To determine the effect of the tested extracts on the DOX-induced toxicity in the H9c2 cardiomyocytes, the cells were treated simultaneously with both DOX and CAS H_2_O or CAS MeOH in a concentration of 125 μg/mL for 48 h. The simultaneous incubation of the 5 µM and 2 µM concentrations of DOX with the CAS H_2_O and CAS MeOH significantly abolished the cytotoxic effect of the DOX on the H9c2 cells. The viability of the H9c2 cells in the case of the combination of DOX and CAS H_2_O or CAS MeOH increased by approximately 40% compared with the DOX administration alone. The obtained results were statistically significant in relation to the DOX and extracts used individually. For further in-depth research, the combination of 2 µM of DOX with CAS H_2_O or CAS MeOH was selected.

**Figure 2:**
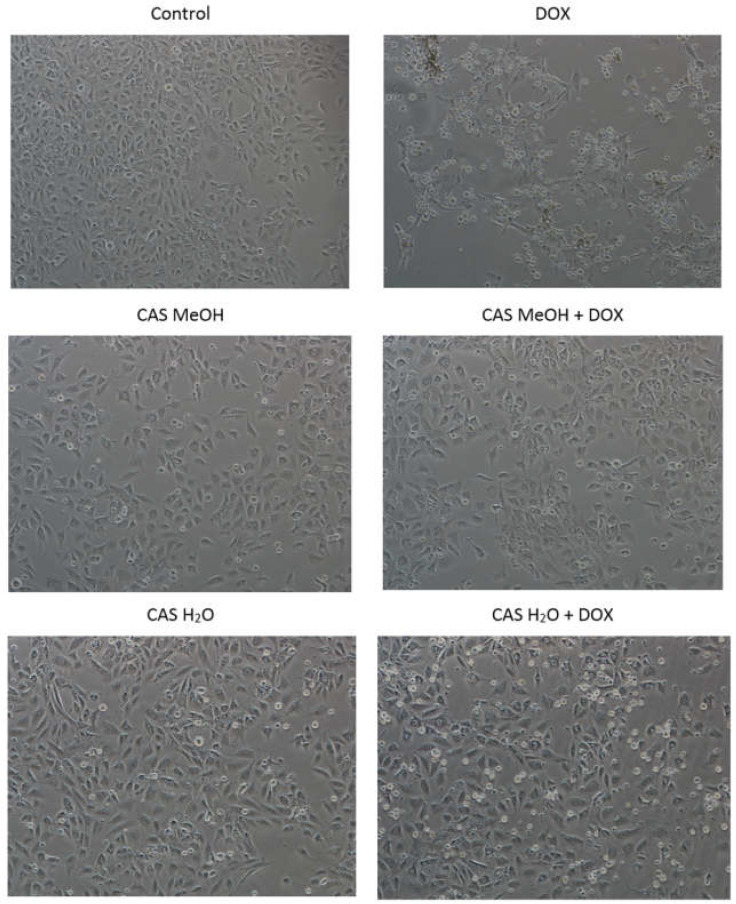
Morphological changes in H9c2 cells. Cells were treated for 48 h with 2 μM of DOX and 125 μg/mL of CAS H_2_O or 125 μg/mL of CAS MeOH, or a combination (DOX + CAS MeOH/DOX + CAS H_2_O) (magnification: ×100). DOX: doxorubicin; CAS MeOH: *Centaurea castriferrei* Borbás & Waisb methanol–water (7:3 *v*/*v*) extract; CAS H_2_O: *Centaurea castriferrei* Borbás & Waisb water extract.

The microscopic observation of the morphology of the H9c2 cardiomyocytes conducted with a Nikon Eclipse Ti phase-contrast microscope confirmed the results obtained in the MTT test. After the DOX treatment, a markedly reduced number of adherent cells was observed in the field of view in comparison with the control cultures (Figure 2). In addition, many cells were shrunken, dead, and detached from the bottom of the culture plate. The H9c2 cells treated with CAS H_2_O or CAS MeOH alone were not different from the control cells.

The simultaneous treatment of the H9c2 cells with the DOX and CAS H_2_O or CAS MeOH extracts revealed a noticeably lower number of dead and shrunken cells compared with the DOX used alone. Additionally, more adherent cells were visible in the field of view.

### 2.3. Effects of CAS Extracts and DOX on Cell Cycle Progression in H9c2 Cells

**Figure 3:**
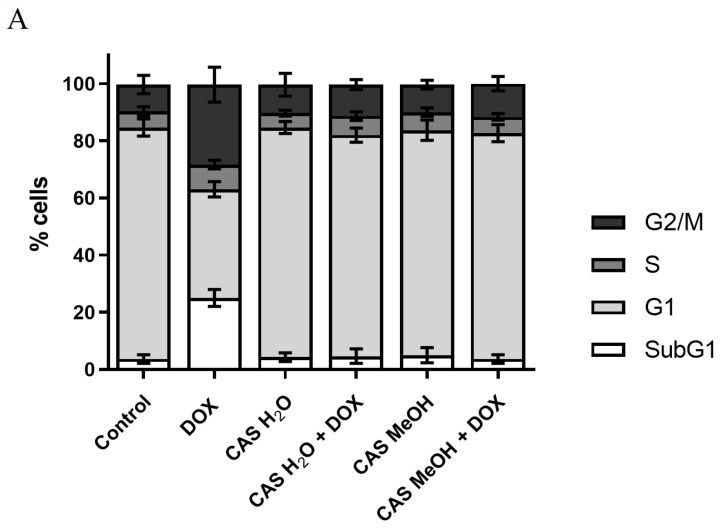

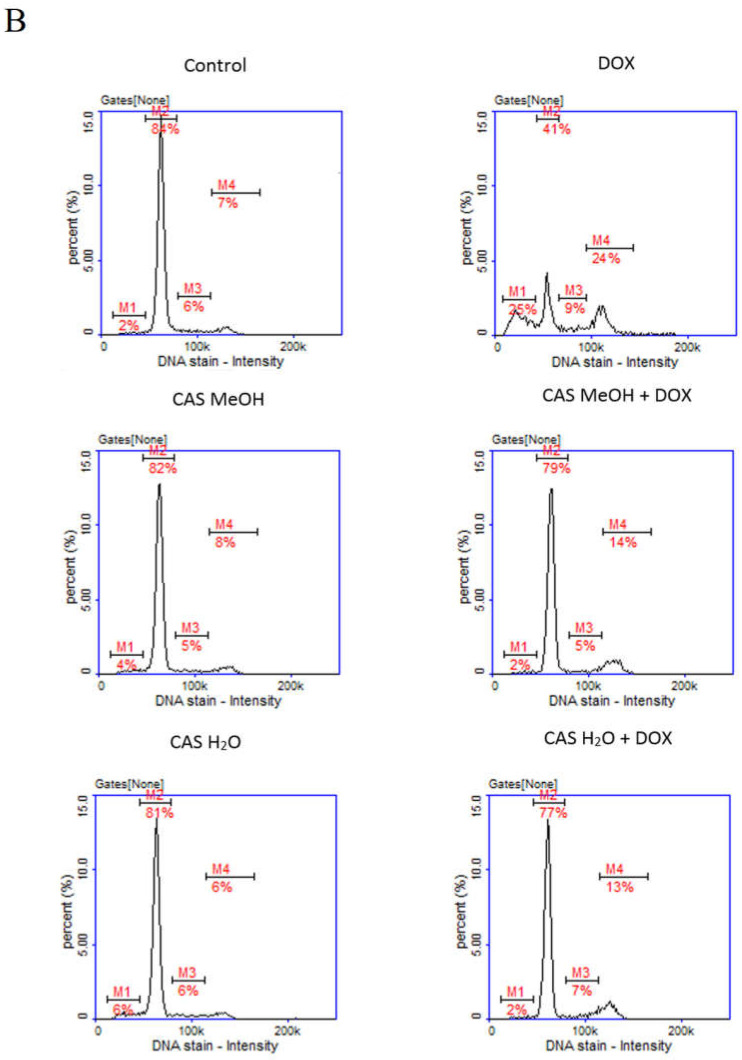
(**A**) Cell cycle analysis of H9c2 cells by image cytometry. Cells were treated for 48 h with 2 μM of DOX and 125 μg/mL of CAS H_2_O or 125 μg/mL of CAS MeOH, or a combination (DOX + CAS MeOH/DOX + CAS H_2_O). Values obtained from three independent experiments are presented as means ± SDs. (**B**) H9c2 cell cycle histograms representative of all repetitions of experiment (M1: subG1 phase; M2: G1 phase; M3: S phase; M4: G2/M phase). DOX: doxorubicin; CAS MeOH: *Centaurea castriferrei* Borbás & Waisb methanol–water (7:3 *v*/*v*) extract; CAS H_2_O: *Centaurea castriferrei* Borbás & Waisb water extract.

The cell cycle analysis showed that the DOX used alone caused a significant increase in the population of the cells in the subG1 phase, which corresponded to dead cells, with a simultaneous decrease in the peak that corresponded to the cells in the G1 phase, compared with the control (Figure 3A,B). Moreover, an elevated percentage of the cells in the G2/M phase was observed, which proved the presence of both cytotoxic (increased subG1 phase) and cytostatic effects. The CAS H_2_O and CAS MeOH alone had no significant effects on the cell cycle, and the achieved results were similar to those of the control sample. The combined treatment of the H9c2 cells with DOX and CAS H_2_O or CAS MeOH contributed to the elimination of the cytotoxic and cytostatic effects that result from the action of the DOX used alone. The percentage of H9c2 cells in each phase of the cell cycle for the combination of the DOX and extract was similar to those obtained in the control cultures.

### 2.4. Effects of CAS Extracts on DOX-Induced Apoptosis in H9c2 Cells

**Figure 4:**
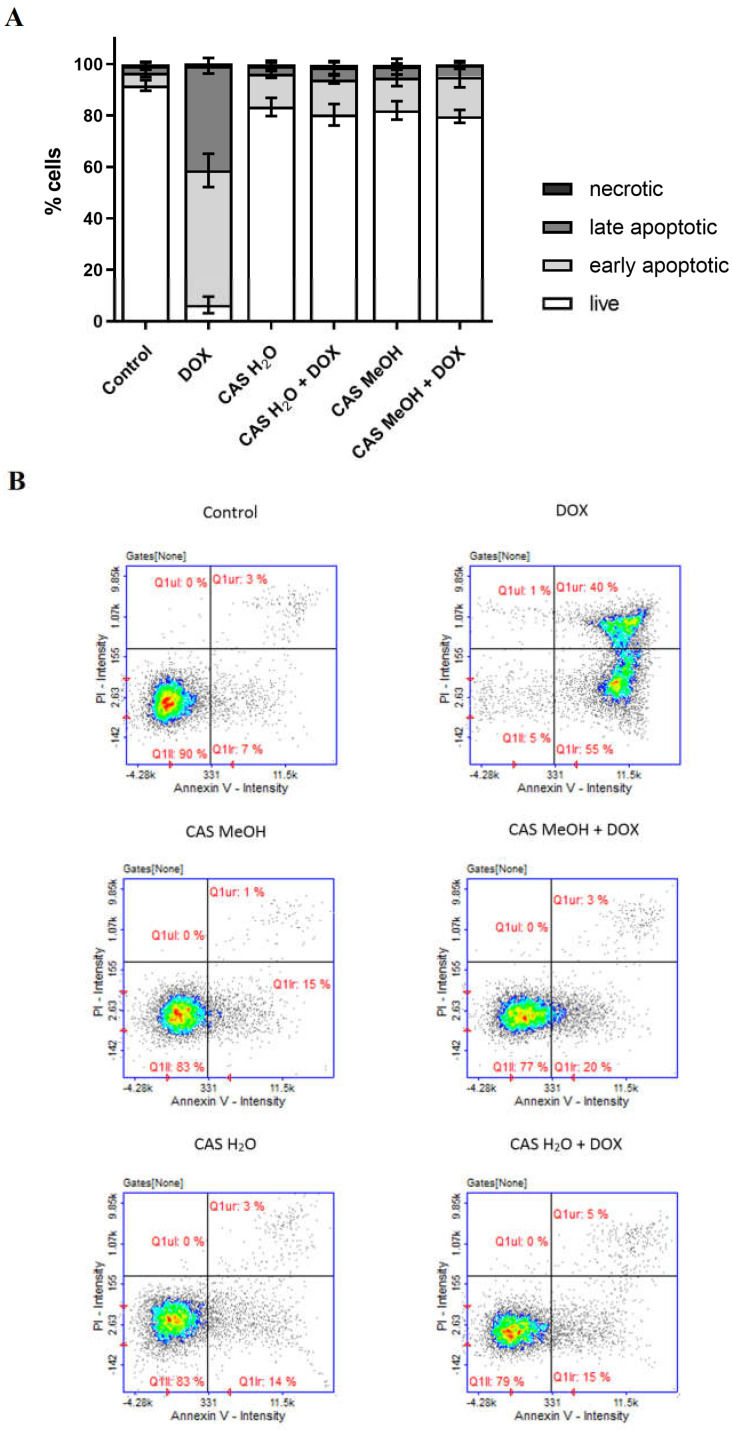
(**A**) Detection of cell apoptosis in H9c2 cells by image cytometry. Cells were treated for 48 h with 2 μM of DOX and 125 μg/mL of CAS H_2_O or 125 μg/mL of CAS MeOH, or a combination (DOX + CAS MeOH/DOX + CAS H_2_O). Values obtained from three independent experiments are presented as means ± SDs. (**B**) Representative histograms (Q1II: live cells; Q1Ir: early apoptotic cells; Q1ur: late apoptotic cells; Q1uI: necrotic cells). DOX: doxorubicin; CAS MeOH: *Centaurea castriferrei* Borbás & Waisb methanol–water (7:3 *v*/*v*) extract; CAS H_2_O: *Centaurea castriferrei* Borbás & Waisb water extract.

The detection of the apoptosis using image cytometry revealed that virtually all the H9c2 cells treated with DOX were in the early and late stages of apoptosis (Figure 4A,B). In the case of the cells incubated with CAS H_2_O or CAS MeOH, only approximately 15% of the cells were early apoptotic. Similar results were obtained in the context of the simultaneous incubation of the DOX and CAS H_2_O or CAS MeOH.

### 2.5. Effects of CAS Extract on DOX-Induced Oxidative Stress in H9c2 Cells

#### 2.5.1. Detection of Oxidative Stress

**Figure 5:**
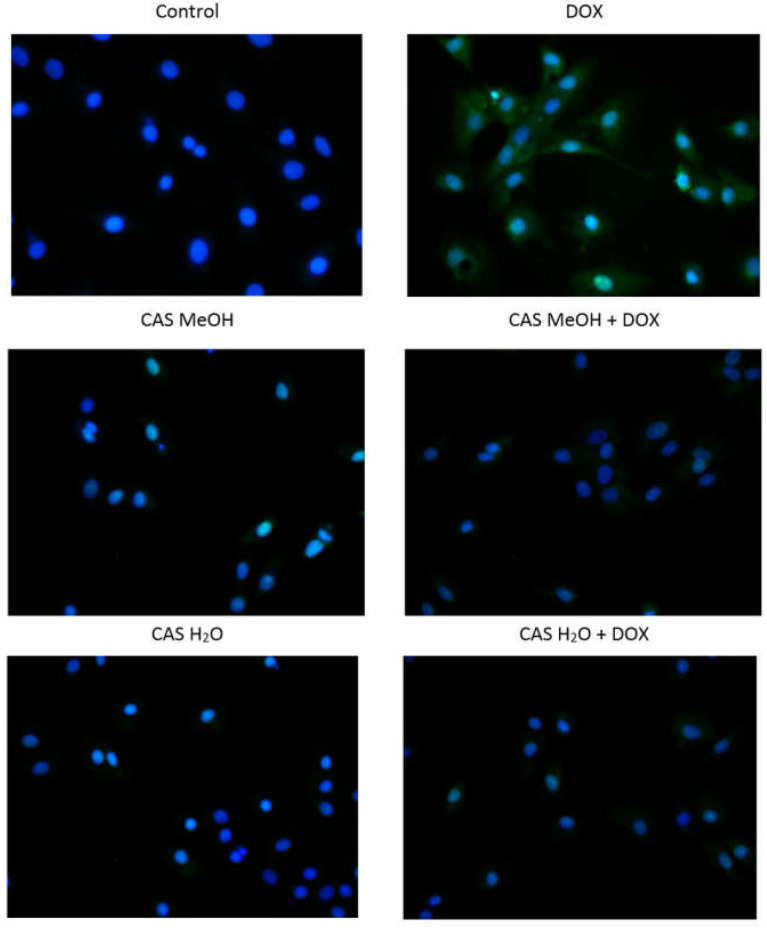
Detection of oxidative stress in H9c2 cells using CellROX Green Reagent. Cells were treated for 48 h with 2 μM of DOX and 125 μg/mL of CAS H_2_O or 125 μg/mL of CAS MeOH, or a combination (DOX + CAS MeOH/DOX + CAS H_2_O) (magnification: ×100). DOX: doxorubicin; CAS MeOH: *Centaurea castriferrei* Borbás & Waisb methanol–water (7:3 *v*/*v*) extract; CAS H_2_O: *Centaurea castriferrei* Borbás & Waisb water extract.

To assess the presence of ROS in the tested H9c2 cells, green fluorogenic probe CellROX Green Reagent was used. In the H9c2 cells incubated with DOX, high signals came from both the nuclei and mitochondria (Figure 5). In the case of the cells treated with CAS MeOH, very faint signals derived from the nuclei were detected. CAS H_2_O does not cause the formation of ROS, which was reflected in the lack of green fluorescence, as in the control cells. The simultaneous treatment with DOX and CAS H_2_O or CAS MeOH led to the abolition of the high nucleus and mitochondrial signals in comparison with DOX alone. The obtained images were similar to those that were obtained from the extracts used individually.

#### 2.5.2. Determination of DNA Oxidative Damage

**Figure 6:**
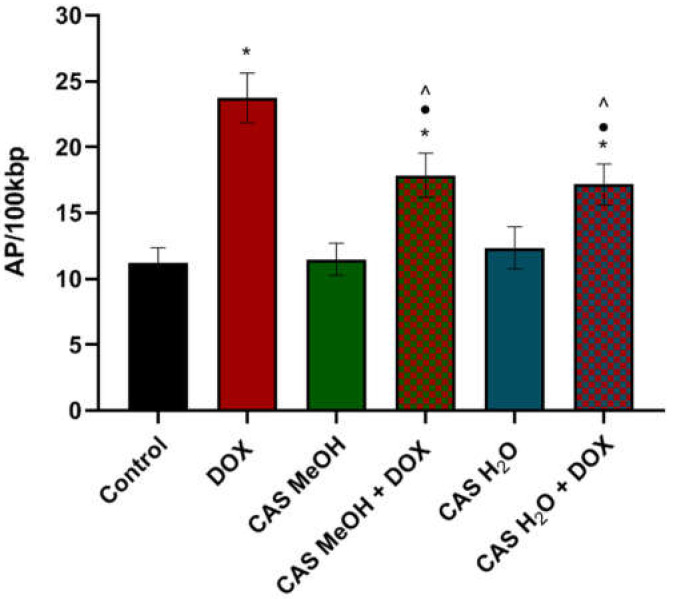
AP site levels in DNA of H9c2 cells. Cells were treated for 48 h with 2 μM of DOX and 125 μg/mL of CAS H_2_O or 125 μg/mL of CAS MeOH, or a combination (DOX + CAS MeOH/DOX + CAS H_2_O). Values obtained from three independent experiments are presented as means ± SDs. * *p* < 0.05 vs. control; • *p* < 0.05 vs. DOX 2 µM; ^ *p* < 0.05 vs. CAS MeOH/CAS H_2_O. DOX: doxorubicin; CAS MeOH: *Centaurea castriferrei* Borbás & Waisb methanol–water (7:3 *v*/*v*) extract; CAS H_2_O: *Centaurea castriferrei* Borbás & Waisb water extract.

The determination of the oxidative DNA damage was carried out in order to assess the oxidative stress influence on the number of AP sites. In the DNA isolated from the H9c2 cells treated with DOX, a statistically significant accumulation of AP sites (23.72 ± 1.90 AP sites/100,000 bp) was observed in comparison with the control cultures (11.23 ± 1.13 AP sites/100,000 bp). In the cases of the CAS H_2_O and CAS MeOH used alone, the numbers of AP sites (12.36 ± 1.59 AP sites/100,000 bp and 11.49 ± 1.22 AP sites/100,000 bp, respectively) were similar to those in the control cultures. However, the simultaneous treatment of the H9c2 cells with DOX and CAS H_2_O or CAS MeOH resulted in statistically significant decreases in the levels of the AP sites (DOX + CAS H_2_O: 7.17 ± 1.55 AP sites/100,000 bp; DOX + CAS MeOH: 17.85 ± 1.68 AP sites/100,000 bp) compared with those of the DOX alone (Figure 6).

#### 2.5.3. Quantitative Real-Time PCR

**Figure 7:**
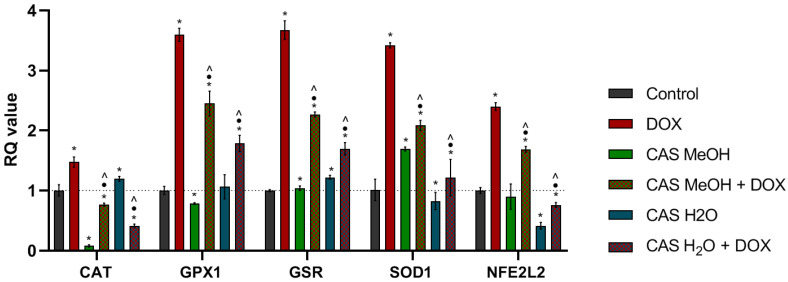
Relative mRNA expression levels of genes related to oxidative stress. *BACT* and *RNA18SN5* were used as reference genes. Results were calculated as RQ values and are presented as means ± SDs. To compare more than two groups, one-way analysis of variance (ANOVA) and post hoc multiple comparisons based on Tukey’s HSD test were used. * *p* < 0.05 vs. control; • *p* < 0.05 vs. DOX 2 µM; ^ *p* < 0.05 vs. CAS MeOH/CAS H_2_O. DOX: doxorubicin; CAS MeOH: Centaurea castriferrei Borbás & Waisb methanol–water (7:3 *v*/*v*) extract; CAS H_2_O: Centaurea castriferrei Borbás & Waisb water extract.

The quantitative real-time PCR was performed to assess the relative expressions of the genes related to the defense against oxidative stress. The analysis revealed that the DOX caused the statistically significant upregulation of all the tested genes in comparison with the control (Figure 7). The smallest increase was noted for *CAT*, which encodes the enzyme catalase. After the CAS MeOH treatment, the *CAT* and *GPX* expressions were downregulated, while the *SOD* and *GSR* expressions were higher than those of the control. The CAS H_2_O caused slight increases in the *CAT* and *GSR* expressions and decreases in the *SOD* and *NFE2L2* expressions. The simultaneous treatment with DOX and CAS H_2_O or CAS MeOH led to the statistically significant downregulation of all the tested genes compared with DOX used alone.

### 2.6. Effects of CAS Extracts and DOX on MCF-7 Breast Cancer Cells

**Figure 8:**
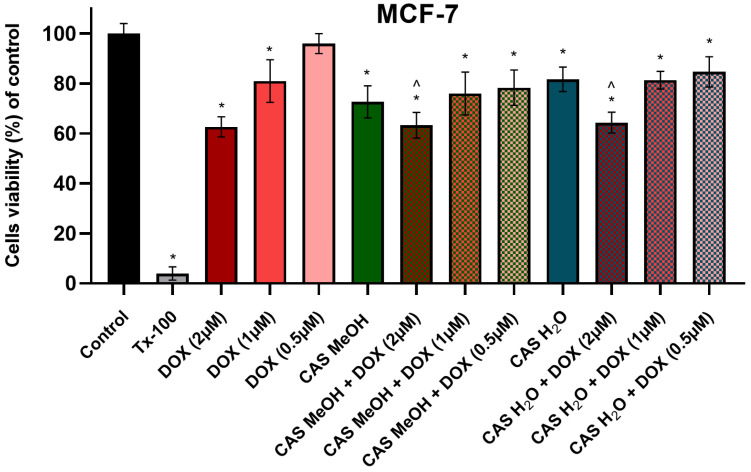
MCF-7 breast cancer cell viability (% of control) based on MTT assay. Cells were treated for 48 h with 2–0.5 μM of DOX and 125 μg/mL of CAS H_2_O or 125 μg/mL of CAS MeOH, or a combination (DOX + CAS MeOH/DOX + CAS H_2_O). In addition, cells were treated with DMSO as a vehicle in control cultures and 1% Tx-100 in media as a positive control for 48 h. The values obtained from three independent experiments are presented as means ± SDs. * *p* < 0.05 vs. control; ^ *p* < 0.05 vs. CAS MeOH/CAS H_2_O. Tx-100: Triton-x100; DOX: doxorubicin; CAS MeOH: *Centaurea castriferrei* Borbás & Waisb methanol–water (7:3 *v*/*v*) extract; CAS H_2_O: *Centaurea castriferrei* Borbás & Waisb water extract.

To assess the effects of the combined treatment with DOX and the tested extracts on the breast cancer cell line (MCF-7), a cytotoxicity analysis was performed using the MTT test. The DOX was used in a wide range of concentrations (2–0.5 µM), and it decreased the viability of the studied MCF-7 cells in a dose-dependent manner. The CAS H_2_O and CAS MeOH used alone had statistically significant cytotoxic effects on the MCF-7 cells (81.66 ± 4.93% and 72.67 ± 6.43%, respectively) in comparison with the control cultures (Figure 8). The simultaneous incubation of every used concentration of DOX with the CAS H_2_O or CAS MeOH did not result in greater cytotoxicity compared with the DOX used alone.

## 3. Discussion

Despite the constant development of new methods of cancer treatment, many therapeutic regimens are based on chemotherapeutics that have been known for years, such as DOX. This anthracycline still represents the treatment base of a wide variety of solid organ tumors and hematological malignancies [25,26]. The main limitation of anthracycline therapy is the cumulative dose-dependent cardiotoxicity, which can lead to irreversible heart failure in patients. Anthracycline cardiomyopathy can occur in patients even years after the discontinuation of the DOX therapy [5,27]. The mechanism of DOX-associated cardiomyopathy is complex and multifactorial. However, the main cause of this phenomenon is the oxidative stress that is associated with the redox cycling of this drug [28,29]. Therefore, scientists are looking for compounds or extracts that can counteract the cardiotoxic effects of DOX. One of the concepts is the combination of DOX with antioxidants that reduce the oxidative stress level.

For many years, the plants of the genus Centaurea L. have been the subject of the research of scientists in many fields of both medicine and pharmacy. Studies on the plant extracts obtained from various species of Centaurea plants have demonstrated their antioxidant, anticancer, and anti-inflammatory properties [17,23,30,31,32]. In the conducted studies, we examined the potential of water and methanol–water extracts from the plant *Centaurea castriferrei* obtained by the UAE method to abolish the cardiotoxic effect of doxorubicin in vitro in H9c2 rat fetal cardiomyocytes. The performed analysis revealed that both the CAS H_2_O and CAS MeOH extracts used at a concentration of 125 µg/mL significantly abolished the cytotoxic effect of DOX (5 and 2 µM) on the H9c2 cells. The abolition of the cytotoxic effect of DOX on cardiomyocytes has also been proven in studies performed by Korga et al. for the case of methanol extracts from two other plant species of the genus Centaurea: *Centaurea borysthenica* and *Centaurea daghestanica* [33]. This may indicate that the plant extracts from this species have the potential to protect cardiomyocytes from the damage caused by DOX. Many studies indicate that plant extracts, in general, may be promising agents for the alleviation of the side effects of DOX use associated with cardiomyocyte damage [34,35,36,37,38].

Studies performed by Aktumsek et al. indicate that all five tested Centaurea species (inter alia, *C kurdica*, *C. rigida*, *C. cheirolopha*, *C. amanicola*, and *C. ptosimmopappoides*) have strong antioxidant properties, and especially methanol extracts [22]. Previous research performed by our group revealed that both the tested extracts have antioxidant activity, but it is higher in the case of CAS MeOH [16]. In order to assess whether the mechanism of abolishing the cardiotoxic effect of DOX by the tested extracts is related to the oxidative stress and antioxidant activities of the extracts, the detection of ROS by the CellROX reagent and the determination of the DNA oxidative damage were performed. The CAS H_2_O and CAS MeOH significantly reduced the DOX-induced oxidative stress in the H9c2 cells. Based on these experiments, it can be concluded that the decrease in the oxidative stress levels in the tested cells by the *C. castriferrei* extracts is one of the mechanisms that is responsible for reducing the cardiotoxic effect of DOX. In the previous research, we determined that both CAS H_2_O and CAS MeOH extracts contain many active compounds, including apigenin and its derivative 7-O-glucuronide [16]. Sahu et al. demonstrated the ability of apigenin to suppress doxorubicin-induced cardiotoxicity via, inter alia, the inhibition of oxidative stress [39]. Another compound that was present in the tested extracts was chlorogenic acid [16]. There are many reports on the strong antioxidant properties of this compound [40,41,42,43,44,45]. Therefore, we suspect that the presence of active compounds, among others from the group of flavonoids and biologically active dietary polyphenols, plays an important role in reducing the levels of oxidative stress in H9c2 cells exposed to DOX [46]. The analysis of the expressions of the genes that are involved in the antioxidant defense system also confirms the mechanism of action that is associated with the reduction in oxidative stress.

Many scientists are cautious about the use of various supplements during anticancer treatment because the administered compounds or extracts may affect the effectiveness of the therapy. Therefore, we conducted a study on the combination of DOX with the tested extracts on the MCF-7 breast cancer cell line to assess the effects of the CAS H_2_O and CAS MeOH on the applied therapy. The analysis indicated that the addition of the tested extracts to any of the tested concentrations of DOX did not abolish the effect of the chemotherapeutic agent. Similar results were obtained in the studies of Korga et al., in which the *Centaurea daghestanica* extract did not affect the DOX cytotoxic activity in multiple myeloma cells, while the *Centaurea borysthenica* extract attenuated the effect of the DOX in the highest concentrations [33]. Furthermore, there are no reports on the combined therapy of Centaurea L. plant extracts and chemotherapeutic agents on the various types of cancer. However, many studies that used various plant extracts indicate the enhancement of the effect of DOX on the tested cancer cells [47,48,49,50,51].

## 4. Materials and Methods

### 4.1. Plant Material

The plant material (flowering herbs of the species *C. castriferrei* from the genus *Centaurea* L.) for the research was obtained from the Botanical Gardens (Medicinal Plant Laboratory) of the Department of Pharmacognosy of the Medical University of Lublin. The plants were harvested in 2020 from June to August. The acquired plant material was dried in a forced-air dryer that did not exceed 30 °C. For the further experimental procedures, the upper parts of the stems, leaves, and flowers were ground in an electric grinder and sieved according to the requirements of Polish Pharmacopoeia XII (2020). The plant material was identified by Aleksandra Józefczyk, PhD. Specimen C. castriferrei-2020 was deposited in the Department of Pharmacognosy.

### 4.2. Extraction of Plant Material by Ultrasound-Assisted Extraction (UAE)

The plant material extracts were prepared using an ultrasonic bath (Sonorex Bandelin, Berlin, Germany). Two types of extractants were used in the preparation of the extracts: distilled water and a mixture of methanol and water (7:3 *v*/*v*). For the extraction, 120 mL of the extractant was added to 20 g of powdered plant material. The extractions were conducted for 30 min at 65 °C. After cooling, the extracts were filtered, and the residual plant material was extracted again under the same conditions. These steps were repeated. The obtained methanol–water extract was distilled to dryness and redissolved in this extract, while the water extract was concentrated. Both extracts were transferred to a 100 mL volumetric flask.

Stock solutions for all biological experiments were prepared by dissolving 50 mg of dry plant extracts in 1 mL of DMSO.

### 4.3. Phytochemical Analysis

#### 4.3.1. RP-HPLC/DAD Analysis

The extracts used in the study were analyzed using an Agilent Technologies 1100 Series liquid chromatograph (Agilent Technologies, Waldbronn, Germany) with a visible diode array detector (DAD) and an autosampler. The analysis was performed in line with previously conducted research [16].

#### 4.3.2. LC/ESI–QTOF–MS Analysis

The polyphenolic components of the used extracts were determined by an extended qualitative analysis carried out by an HPLC/ESI–QTOF–MS system in the negative ion mode using a 6530B Accurate-Mass QTOF-LC/MS (Agilent Technologies Inc., Santa Clara, CA, USA) mass spectrometer with an ESI-jet stream ion source. The analysis was performed in line with previously conducted research [16].

### 4.4. Cell Culture and Treatment

The research was performed on adherent rat embryonic cardiomyocytes: H9c2 and the breast cancer cell line MCF-7 (ATCC, Manassas, VA, USA). The cell lines were cultured in Dulbecco’s Modified Eagle’s Medium (DMEM) and Eagle’s Minimum Essential Medium (EMEM) (Corning, New York, NY, USA), respectively. The culture media were supplemented with 10% fetal bovine serum (Life Technologies, Carlsbad, CA, USA) and antibiotics: penicillin (100 units) and streptomycin (100 μg/mL) (Sigma-Aldrich, St. Louis, MO, USA). The cells were incubated at 37 °C in 5% CO_2_ in air.

The H9c2 cells were treated for 48 h with 5 μM and 2 μM of the DOX and 125 μg/mL of the water or methanol–water *C. castriferrei* extracts, or a combination (DOX + CAS H_2_O/CAS MeOH). For further in-depth studies, the 2 μM concentration of the DOX was selected. In the cytotoxicity analysis, the MCF-7 breast cancer cells were incubated for 48 h with DOX at concentrations of 2 µM, 1 µM, and 0.5 µM, and with the tested extracts at a concentration of 125 µg/mL, or a combination. DMSO was used as the vehicle in the control cultures. The positive control represents the incubation of cells with the 1% Triton-x100 (Tx-100) containing the medium. The concentrations of DOX were selected based on previous studies using H9c2 cells [52,53].

### 4.5. Cytotoxicity Analysis

The cytotoxicity analysis of the DOX-tested extracts or their combination on the H9c2 and MCF-7 cells was evaluated using the MTT assay (MTT Cell Proliferation Assay Kit- Invitrogen, Waltham, MA, USA). The test is based on the ability of the metabolically active viable cells to convert the orange tetrazolium salt (3-(4,5-dimethylthiazol-2-yl)-2,5-diphenyltetrazolium bromide) to a water-insoluble purple formazan product. Both the used cell lines were seeded into 96-well plates at a concentration of 1.5 × 10^5^ cells/mL, and they were cultured to reach 70–80% confluence. The stock solutions for the cytotoxicity analysis were prepared by dissolving 50 mg of dry plant extracts in 1 mL of DMSO. After a 48 h incubation period with the tested DOX and extracts, the MTT solution (0.5 mg/mL in phosphate-buffered saline) was added to each well with tested cells. Following 4 h of incubation, the MTT solution was removed, and the formed formazan crystals were dissolved in DMSO. The absorbance of the solutions was measured spectrophotometrically at 570 nm with the PowerWave XS microplate spectrophotometer (BioTek Instruments, Winooski, VT, USA). Each assay was evaluated three times and measured in triplicate [54,55].

For the observation of the changes in the morphology of the examined cells, a Nikon Eclipse Ti phase-contrast microscope using NIS-Elements Imaging software (Nikon, Tokyo, Japan) was used.

### 4.6. Cell Cycle Analysis

The cell cycle was evaluated using the NucleoCounter NC-3000 (ChemoMetec, Allerod, Denmark), in compliance with the two-step Cell Cycle Assay protocol (ChemoMetec, Allerod, Denmark). Following 48 h of incubation, the cells were detached from the 6-well plate using a trypsin–EDTA solution (Corning, New York, NY, USA), and they were thoroughly resuspended in a 250 μL lysis buffer (Solution 10) supplemented with 10 μg/mL DAPI. The incubation was carried out for 5 min at 37 °C in the dark. Next, 250 μL of stabilization buffer (Solution 11) was added, and the suspension was loaded onto an 8-chamber slide (NC-Slide A8, Chemometec., Allerod, Denmark). The results were analyzed in the NucleoCounter NC-3000. Each experiment was carried out three times with three replicates [56,57,58].

### 4.7. Apoptosis Detection

The apoptosis detection was performed using the NucleoCounter NC-3000 (ChemoMetec, Allerod, Denmark), in compliance with the Annexin V Apoptosis Assay protocol (ChemoMetec, Allerod, Denmark). After 48 h of incubation, the cells were detached from the 6-well plate using a trypsin–EDTA solution, and they were stained with Annexin V–FITC (fluorescein isothiocyanate), Hoechst 33342, and propidium iodide (PI), in accordance with the manufacturer’s recommended protocol. Then, the stained cells were immediately analyzed using 2-chamber NC-Slides A2 in the NucleoCounter NC-3000. Each experiment was carried out three times with three replicates [59,60,61].

### 4.8. Detection of Oxidative Stress

The detection of the ROS in the cells was performed using the CellROX Green Reagent (Invitrogen, Waltham, MA, USA), which is a fluorogenic probe. This reagent is weakly fluorescent when in a reduced state, and it exhibits bright green photostable fluorescence upon oxidation by ROS and the subsequent binding to DNA, with an absorption/emission maxima of 485/520 nm. Following 48 h of incubation, the cells were fixed with 4% PFA and stained with 5 μM of the CellROX Green Reagent and Hoechst 33342 (5 μg/mL) by adding the probe to PBS. Next, the cells were incubated at 37 °C for 30 min. After the incubation period, the cells were washed with PBS three times and imaged on a Nikon Eclipse Ti inverted microscope using a 20× objective with NIS-Elements Imaging software (Nikon, Tokyo, Japan) [62,63,64].

### 4.9. Determination of DNA Oxidative Damage

The determination of the DNA oxidative damage was conducted using a DNA Damage Quantification Kit (Dojindo, Kumamoto, Japan), and by measuring the quantity of the abasic (AP) sites, in compliance with the manufacturer’s protocol. Following 48 h of incubation, the DNA was isolated with the Syngen DNA Mini Kit (Syngen, Wroclaw, Poland), in accordance with the manufacturer’s protocol. The concentration and purity of the genomic DNA were measured using the MaestroNano Micro-Volume Spectrophotometer (Maestrogen Inc., Taiwan), and they were adjusted to 100 μg/mL in the TE buffer. The main cause of the oxidative damage to DNA is the interaction with ROS. ROS oxidative attacks on the deoxyribose moiety in DNA lead to the release of free bases, which causes strand breaks with various sugar modifications and simple abasic sites. An aldehyde-reactive probe (ARP) (N′-aminooxymethylcarbonylhydrazin-D-biotin) reacts specifically with an aldehyde group that is present on the open ring form of AP sites, which makes it possible to detect the DNA modifications that result in the formation of the aldehyde groups. The biotin–avidin-specific connection and horseradish peroxidase were used for the colorimetric detection at 650 nm using a PowerWave™ microplate spectrophotometer (BioTek Instruments, Winooski, VT, USA).

### 4.10. Quantitative Real-Time PCR Analysis (qRT-PCR)

The cells were seeded into 25 cm^3^ flasks at a concentration of 4 × 10^4^ cells/mL, while the imparted compounds were added after reaching 70–80% confluence. After 48 h of incubation, 1 mL of TRIzol™ reagent (Invitrogen, Carlsbad, CA, USA) was added to the cells for lysis. The resulting lysates were centrifuged for 15 min at 12,000× *g* at 4 °C, and the resulting clear supernatants were processed according to the method of Chomczynski and Sacchi [65]. Reverse transcription was then performed using the NG dART RT-PCR kit (EURx, Gdansk, Poland) and a mastercycler gradient thermocycler (Eppendorf, Hamburg, Germany), maintaining the reaction thermal profile: 10 min at 25 °C, followed by 50 min at 50 °C and 5 min at 85 °C.

The qPCR reaction was performed in triplicate, according to the manufacturer’s instructions, using Fast SG/ROX qPCR Master Mix (2×) (EURx, Gdansk, Poland) in a 7500 fast real-time PCR system (ThermoFisher, Waltham, MA, USA). The reference genes were 18SRNA and BACT, and the relative expressions of the tested genes were determined by qRT-PCR and ΔΔCt (Figure 4). A statistical analysis was then performed using the RQ (relative quantification) values (RQ = 2 ^−ΔΔCt^), which were used for the statistical analysis [66,67].

The primers used in the gene expression evaluation are presented in Table 4.

### 4.11. Statistical Analysis

The results are presented as means ± SDs, and they were analyzed with STATISTICA 13 software (StatSoft, Krakow, Poland). For comparing more than 2 groups of means, the one-way analysis of variance (ANOVA) and post hoc multiple comparisons on the basis of Tukey’s HSD test were used. The results were considered statistically significant if the *p*-value was less than 0.05.

## 5. Conclusions

Scientists are still searching for compounds and extracts that reduce the side effects of the chemotherapeutic drugs used in cancer treatment, including the cardiotoxicity of DOX. Studies have indicated that both the water and methanol–water extracts obtained from the plant *Centaurea castriferrei* have great potential to protect cardiomyocytes from the detrimental effect of DOX. Furthermore, the evidence that the tested extracts cause significant reductions in oxidative stress opens the way for further in-depth research. In future studies, it would be valuable to isolate the active compounds that occur in the tested plant extracts and assess their biological activities. A comparison of the potentials of the extracts and their individual active ingredients to abolish the cardiotoxic effects of DOX could be an interesting area of further research.

## Figures and Tables

**Table 1:** Identification and quantification (mg/g dry weight (d.w.)) of contents of main components of tested CAS MeOH and CAS H_2_O *C. castriferrei* extracts by RP-HPLC/DAD analysis.

Main Components	CAS MeOH			CAS H_2_O		
	Content (mg/g)			Content (mg/g)		
		±SD	±RSD		±SD	±RSD
Neochlorogenic acid	0.90	0.01	0.6	0.51	0.00	0.2
Chlorogenic acid	4.14	0.03	0.7	1.30	0.01	0.5
Cryptochlorogenic acid	0.11	0.00	1.7	0.04	0.00	0.0
Caffeic acid	0.09	0.00	0.7	0.09	0.00	1.0
Protocatechuic acid	0.26	0.01	3.5	0.17	0.00	2.2
Chlorogenic acid glucoside	1.70	0.00	0.1	0.14	0.00	0.9
Caffeic acid derivative	0.16	0.00	1.4	0.08	0.00	0.4
Apigenin derivative	0.40	0.01	1.3	0.21	0.00	0.2
Luteolin 7-O-glucoside	0.85	0.01	0.6	0.51	0.01	2.4
Apigenin 7-O-glucoside	0.17	0.00	0.4	None	-	-
Apigenin 7-O-glucuronide	5.26	0.02	0.3	1.76	0.01	0.3
Dimethylapigenin	0.56	0.00	0.3	0.11	0.00	0.6
Dihydrokaempferol	0.82	0.02	0.0	0.23	0.00	0.0
Kaempferol dihydroglucoside	0.09	0.00	0.0	None	-	-
Kaempferol glucoside	0.08	0.00	0.0	None	-	-
Centaurein	3.97	0.02	0.4	0.16	0.00	0.6
Jacein	1.21	0.00	0.2	None	0	0
Apigenin	7.32	0.03	0.4	0.38	0.01	2.6
Luteolin	0.13	0.00	0.0	None	-	-

**Table 2:** CAS MeOH extract. Fragmentation analysis of identified compounds by LC/(-)ESI–QTOF–MS.

No.	Name of Compound	T_R_ (min)	Molecular Ion (M − H) (*m*/*z*)	MS/MS Fragments (*m*/*z*)
1	Chlorogenic acid	15.735	353.0846	191.0542
2	Feruloquinic acid	20.158	367.0989	191.0531; 134.0258; 93.0413
3	Apigenin glucuronide-glucoside	21.429	607.1286	431.0946; 269.0409; 175.0196; 113.0224
4	Kaempferide glucoside	23.376	463.0861	301.0397; 151.0012; 97.3310
5	Isorhamnetin glucoside	24.507	477.1000	315.0631
6	Chlorogenic acid glucoside	25.911	515.1150	353.0852; 191.0536
7	Isorhamnetin glucuronide	26.546	491.1154	315.0631
8	Apigenin glucuronide	27.153	445.0736	269.0441; 175.0241; 113.0202
9	Centaurein	27.455	521.1231	506.1033; 343.0375
10	Jacein	27.960	521.1240	506.1076; 359.0687; 343.0444
11	Hispidulin glucuronide	28.282	475.0842	299.0494; 284.0258; 255.0054; 227.0327; 85.0249
12	Isorhamnetin	30.863	315.0470	300.0327; 199.0447; 65.0458
13	Luteolin	31.090	285.0475	133.0242; 107.0099
14	Apigenin	34.074	269.0310	117.0348
15	Hispidulin	35.317	299.0524	283.0272; 255.0443; 227.0484

**Table 3:** CAS H_2_O extract. Fragmentation analysis of identified compounds by LC/(-)ESI–QTOF–MS.

No.	Name of Compound	T_R_ (min)	Molecular Ion (M − H) (*m*/*z*)	MS/MS Fragments (*m*/*z*)
1	Quinic acid	1.865	191.0525	111.0543
2	Protocatechuic acid glucoside	7.799	153.0165	153.0154; 109.0277
3	Chlorogenic acid	10.087	353.0863	191.0532; 179.0320
4	Neochlorogenic acid	15.646	353.4838	191.0520; 135.0389; 85.0277
5	Feruloylquinic acid	20.186	367.0987	191.0571; 134.0351; 93.0317
6	Ferulic acid	25.478	193.0472	133.0283
7	Apigenin glucuronide	27.153	445.0736	269.0440; 175.0241; 113.0202
8	Apigenin	34.138	269.0439	117.0334

**Table 4:** qPCR primers used in experiment.

Gene Symbol	Gene Name	Forward Sequence (5′→3′)	Reverse Sequence (5′→3′)
*CAT*	Catalase	GCTCCGCAATCCTACACCAT	GGACATCGGGTTTCTGAGGG
*GPX1*	Glutathione peroxidase 1	CAATCAGTTCGGACATCAGGAGA	TAAAGAGCGGGTGAGCCTTC
*GSR*	Glutathione-disulfide reductase	CAAGGAGAAGCGGGATGCTT	ACTTCGATGTGGGACTTGGTT
*SOD1*	Superoxide dismutase 1	GGCCGTACTATGGTGGTCC	CCAATCACACCACAAGCCAAG
*NFE2L2*	NFE2-like bZIP transcription factor 2	CTACAGTCCCAGCAGGACAT	GCAAGCGACTGAAATGTAGGTG
*RNA18SN5*	18S ribosomal N5	GAAACTGCGAATGGCTCATTAAA	CACAGTTATCCAAGTGGGAGAGG
*BACT*	Beta-actin	AGAGCTACGAGCTGCCTGAC	AGCACTGTGTTGGCGTACAG

## Data Availability

The data presented in this study are available upon request from the corresponding author.

## Sample Availability

Samples of the compounds are not available from the authors.
